# Duodenojejunal intussusception secondary to primary gastrointestinal stromal tumor: A case report

**DOI:** 10.1016/j.ijscr.2019.09.041

**Published:** 2019-09-30

**Authors:** Goshi Fujimoto, Shunichi Osada

**Affiliations:** Department of Gastroenterological Surgery, Ofuna Chuo Hospital, Postal address: 6-2-24, Ofuna, Kamakura, Kanagawa 247-0056, Japan

**Keywords:** CT, computed tomography, GIST, gastrointestinal stromal tumor, HPF, high-power field, LR, limited resection, PD, pancreaticoduodenectomy, SMA, smooth muscle actin, Gastrointestinal stromal tumor, Intussusception, Duodenectomy, Case report

## Abstract

**Introduction:**

Gastrointestinal stromal tumors (GISTs) in the third portion of the duodenum are rare. Intussusception and obstruction are rarely caused by GISTs because of their tendency to grow in an extraluminal manner. Herein, we report a case involving segmental duodenectomy in a patient with duodenojejunal intussusception secondary to a primary GIST.

**Presentation of case:**

A 91-year-old woman with a history of iron-deficiency anemia presented with vomiting and anorexia. Preoperative imaging suggested duodenojejunal intussusception secondary to a GIST in the third portion of the duodenum. Segmental duodenectomy with end-to-end duodenojejunostomy without reduction of the intussusception was performed. At 6 months after the surgery, the patient’s anemia had improved and she had no abdominal symptoms.

**Discussion:**

Adult intussusception requires surgical resection because most of the patients have intraluminal lesions. The location in relation to the Vater papilla, tumor size, and resection margin should be considered when selecting the type of surgical resection for duodenal GIST. Limited resection appears to be better than pancreaticoduodenectomy with respect to postoperative complications. Considering the age and performance status of this patient, a less invasive maneuver was selected.

**Conclusion:**

Duodenal GISTs can be a rare cause of intussusception. Thus, a limited surgical resection procedure should be considered in such cases.

## Highlights

• Gastrointestinal stromal tumor (GIST) in the third portion of the duodenum is rare.
• Intussusception and obstruction caused by GIST are uncommon.
• Adult intussusception usually requires surgical resection.
• If the location, tumor size, and resection margin of duodenal GIST are adequate for limited resection, it seems to be better than pancreaticoduodenectomy.
• Elderly patients can have improved quality of life with minimally invasive surgery.

## 1 Introduction

A gastrointestinal stromal tumor (GIST) is defined as a spindle, epithelioid, or occasionally pleiomorphic mesenchymal tumor of the gastrointestinal tract that expresses the KIT protein [1,2], and approximately 2% of all neoplasms of the gastrointestinal tract are classified as GISTs [3]. GISTs are most commonly located in the stomach (60–70%) and rarely in the duodenum [1]. Duodenal GISTs comprise 1–5% of all GISTs and most commonly arise in the second portion of the duodenum, followed by the third, fourth, and first portions [[4], [5], [6]]. Most tumors located at the angle of Treitz are GISTs [7].

Bleeding has been reported to be the most common presenting symptom of GIST, followed by the presence of abdominal mass, intestinal obstruction, and biliary obstruction [5,8]. Since intestinal GISTs tend to grow in an extraluminal fashion, they rarely cause intussusception [3,8].

Herein, we report a case involving segmental duodenectomy in a patient with duodenojejunal intussusception secondary to GIST in the third portion of the duodenum. This work has been reported in line with the SCARE criteria [9].

## 2 Presentation of case

**Fig. 1:**
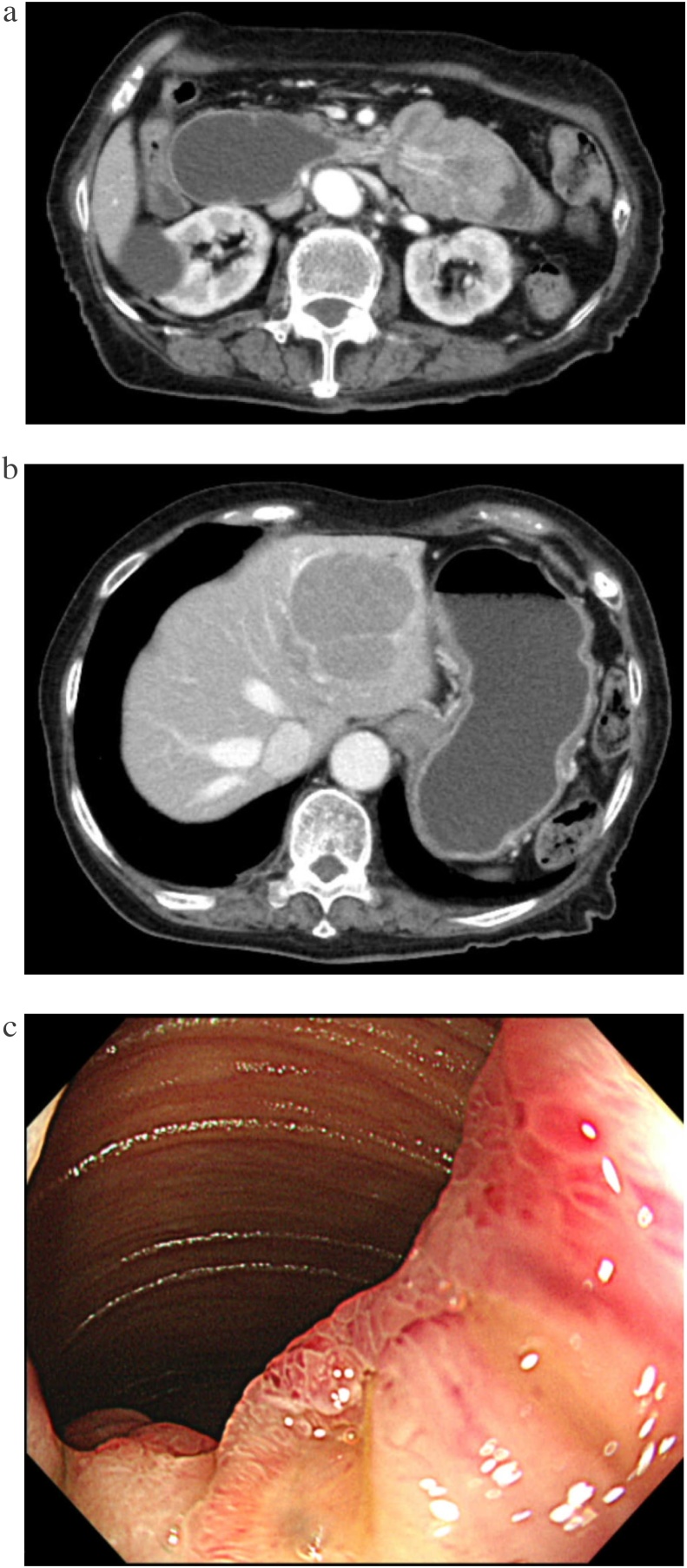
**a**) Findings on contrast-enhanced computed tomography. Intussusception of the third portion of the duodenum is seen, as is duodenal dilation on the oral side of the lesion in the arterial phase. **b**) Findings on computed tomography in the equilibrium phase. Two masses without exophytic growth in the left hepatic lobe are seen, suggesting metastasis. **c**) Findings on esophagogastroduodenoscopy. A protruding lesion in the third portion of the duodenum is seen, which the scope is able to pass through.

A 91-year-old woman with a history of breast cancer, cholecystitis, ascending colon cancer, and iron deficiency anemia presented with vomiting and anorexia. Her body mass index was 23.4 kg/m^2^. Her abdomen was soft and flat with previous operative right pararectal and right subcostal incision scars. On blood examination, her hemoglobin and albumin levels were low (9.2 and 2.7 g/dL, respectively) (Table 1). Contrast-enhanced computed tomography (CT) showed a mass in the third portion of the duodenum presenting as intussusception (Fig. 1a). Two masses without exophytic growth in the left hepatic lobe in the equilibrium phase of contrast-enhanced CT were also observed, suggesting metastasis (Fig. 1b). Esophagogastroduodenoscopy revealed a protruding lesion in the third portion of the duodenum, with subsequent biopsy confirming that it was a GIST (Fig. 1c).

**Table 1:** Blood examination results of the patient.

**Complete blood count**		**Serum chemistry**			
WBC	4750/μL	TP	5.1 g/dL	ALP	263 IU/L
RBC	326 × 10^4^/μL	Alb	2.7 g/dL	γGTP	32 IU/L
Hb	9.2 g/dL	T-Bil	0.5 mg/dL	AMY	55 IU/L
Ht	30.3%	D-Bil	0.2 mg/dL	Na	135 mEq/L
Plt	19.6 × 10^4^/μL	BUN	15 mg/dL	K	4.1 mEq/L
		Cr	0.49 mg/dL	Cl	106 mEq/L
**Blood coagulation test**		LDH	155 IU/L	CRP	0.55 mg/dL
PT (INR)	1.1	CK	12 IU/L	CEA	1.5 ng/dL
PT	83.3%	AST	40 IU/L	CA15-3	4.6 U/mL
APTT	31.6 s	ALT	20 IU/L		

Alb, albumin; ALP, alkaline phosphatase; ALT, alanine aminotransferase; AMY, amylase; APTT, activated partial thrombin time; AST, aspartate aminotransferase; BUN, blood urea nitrogen; CA15-3, carcinoma antigen 15-3; CEA, carcinoembryonic antigen; CK, creatine kinase; Cl, chlorine; Cr, creatinine; CRP, C-reactive protein; D-Bil, direct bilirubin; γGTP, γ-glutamyltransferase; Hb, hemoglobin; Ht, hematocrit; INR, international normalized ratio; K, potassium; LDH, lactate dehydrogenase; Na, sodium; Plt, platelet; PT, prothrombin time; RBC, red blood cell; T-Bil, total bilirubin; TP, total protein; WBC, white blood cell.

**Fig. 2:**
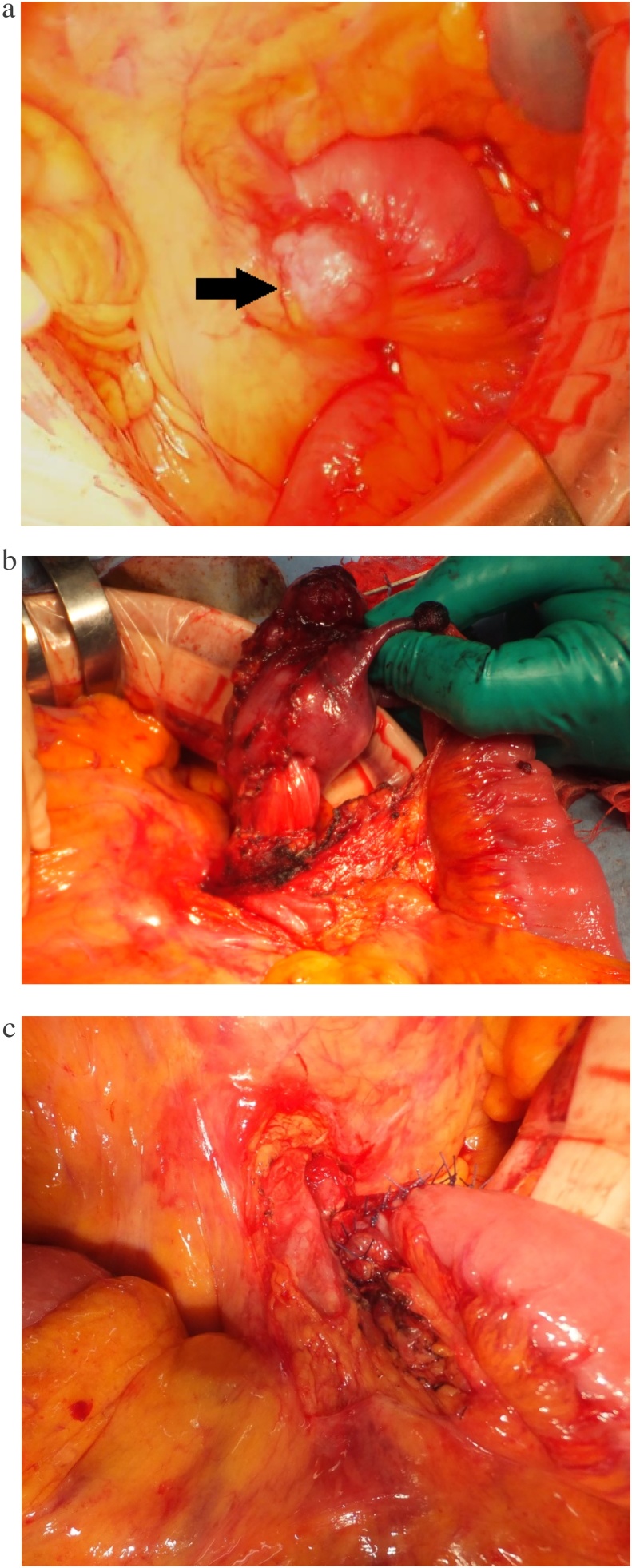
**a**) Findings during laparotomy. Lymph node metastasis (arrow) adjacent to the ligament of Treitz is seen. **b**) Findings during laparotomy after resection of the ligament of Treitz. Duodenojejunal intussusception secondary to gastrointestinal stromal tumor in the third portion of the duodenum was confirmed. **c**) Anastomosis. End-to-end duodenojejunostomy was performed on the left side of the superior mesenteric blood vessels.

Considering her age and physical status, we planned to resect only the duodenum unless the metastatic liver tumor had subcapsular localization and a risk of rupture. Laparotomy revealed lymph node metastasis, but no peritoneal metastasis and no exposure of the liver tumors to the abdominal cavity was observed (Fig. 2a). The ligament of Treitz was resected, allowing for visualization of the duodenojejunal intussusception (Fig. 2b). After the GIST and third potion of the duodenum were dissected from the pancreas, segmental duodenectomy with end-to-end duodenojejunostomy without reduction of the intussusception was performed on the left side of the superior mesenteric blood vessels (Fig. 2c). Two swollen lymph nodes interfering with anastomosis were resected. The total operating time was 2 h 34 min, and the total intraoperative blood loss was 207 mL. The patient had no postoperative complications except for surgical site infection.

**Fig. 3:**
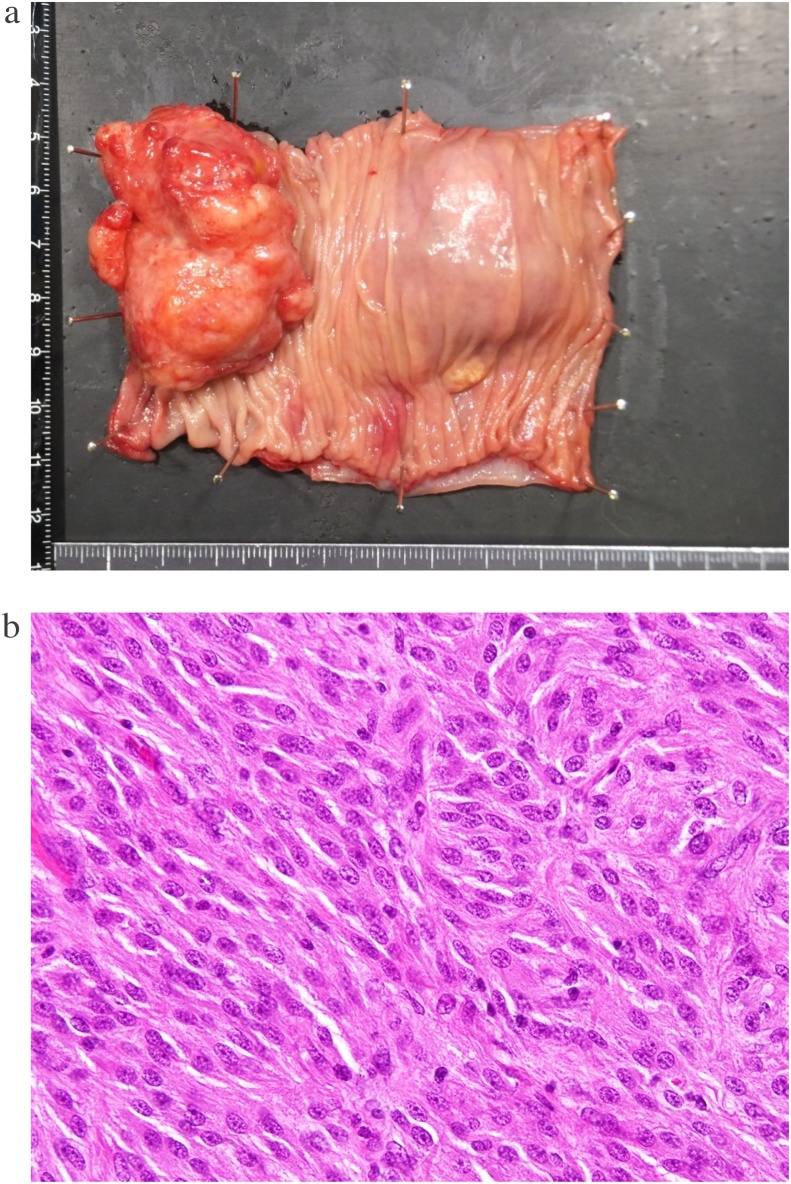
**a**) Macroscopic findings. The specimen shows a smoothly marginated tumor measuring 40 × 40 × 15 mm with an erosive lesion on the mucosal side. **b**) Microscopic pathologic findings (hematoxylin & eosin staining). The specimen shows epithelioid/spindle tumor cells with oval nuclei, which were positive for CD117, DOG1, and CD34.

The resected specimen showed a smoothly marginated tumor measuring 40 × 40 × 15 mm with an erosive lesion on the mucosal side and two elastic soft tumors on the serosal side (Fig. 3a). Histologic examination revealed epithelioid/spindle tumor cells with oval nuclei, which were positive for CD117, DOG1, and CD34 (Fig. 3b). Thus, the tumor was diagnosed as GIST accompanied by lymph node metastasis. The Ki-67 index was 8% and the mitotic count was 20 per 50 high-power fields (HPFs). At 6 months after surgery, the patient reported no abdominal symptoms and her anemia had improved, with a hemoglobin level of 15.3 g/dL.

## 3 Discussion

In this report, we described the diagnosis and management strategies in a rare case involving duodenojejunal intussusception secondary to primary GIST in the third portion of the duodenum. Although gastroduodenal, jejunoileal, and ileocolic intussusceptions have been reported, to our knowledge, this is the first report of a patient with duodenojejunal intussusception secondary to GIST [[10], [11], [12]]. Since GISTs tend to displace adjacent structures without invading them, they can grow to large sizes before symptoms appear. Most duodenal GISTs are accompanied by ulceration of the mucosa, which helps to detect the tumor on endoscopic examination. On abdominal ultrasonography, the presence of a large (>4 cm) tumor with irregular extraluminal borders, echoic foci, and cystic spaces suggests malignancy [2]. Percutaneous fine-needle aspiration is not recommended because of the risk of intra-abdominal tumor dissemination [2,13].

In adults, intestinal invagination or intussusception is rare, accounting for 5% of all intussusceptions and 1% of all intestinal obstructions [14]. There is surgical consensus that adult intussusception requires surgical resection because a majority of patients have intraluminal lesions [14]; adult colonic intussusception should be resected en bloc. It is controversial whether initial reduction should be performed prior to resection [14]; the risks of intraluminal seeding, venous embolization in regions of ulcerated mucosa, and anastomotic complications should be considered in cases of initial reduction [14].

In this case, the preoperative diagnosis was intussusception accompanied by duodenal GIST; thus, we planned limited resection (LR). The Cattell-Braasch maneuver, which permits the surgeon to elevate the right colon and entire small bowel cephalad, was considered in order to observe the third portion of the duodenum completely if the GIST had been located behind the superior mesenteric vessels and transverse mesocolon [15]. However, we did not have to use the Cattell-Braasch maneuver, and segmental duodenectomy without reduction of the intussusception was performed. If the GIST had been located in the second portion of the duodenum, we might have performed pancreaticoduodenectomy (PD). However, LR is reported to have less postoperative complications, better disease-free survival, and lower rate of distant metastasis than PD [5,6]. Although these reports have selection bias, LR seems to be better in selected cases because there is no difference in recurrence between LR and PD [5]. The location in relation to the Vater papilla, tumor size, and a 1- to 2-cm resection margin should be considered when selecting the type of surgical resection [5,16]. Injury to the GIST capsule should be avoided, and adjacent organs should be resected if the GIST invades them [5,16].

The peritoneum and liver are the most common sites of metastasis, whereas regional lymph node metastasis is rare (0–10%), except in Carney’s triad [2,5,8,13,17]. Therefore, the necessity of regional lymph node resection is unknown, and extensive lymphadenectomy is not recommended [2,13,16]. In this case, only two lymph nodes interfering with anastomosis were resected with caution for vascular injury, which can lead to an increase in resection range. The synchronous metastatic liver tumors, which are rarely reported to rupture spontaneously, were not resected considering her age and physical status. The risk of spontaneous rupture of the tumors seemed low because they did not have exophytic growth or subcapsular localization [18,19]. Laparoscopic resection of GIST has been reported, but the oncologic integrity is unknown [13].

Immunohistochemical staining for CD117 (c-kit), CD34, desmin, smooth muscle actin (SMA), and S100 should be performed [2]. The positivity rate for CD117 is approximately >95%; CD34, 60–70%; desmin, 1–2%; SMA, 20–30%; and S100, approximately 5% [[1], [2], [3]]. As not only GISTs but also angiosarcomas and metastatic melanomas are often kit-positive, GISTs must be differentiated from the other tumors using other markers [1]. DOG1 activity is observed in 87% of GISTs and in 79% of GISTs with PDGFRA mutations, whereas CD117 is observed in 74% and 9%, respectively [3,20]. P16 loss and the Ki-67 index, which are both indicated as negative prognostic factors, are lower in duodenal GISTs than in gastric and small bowel GISTs. The oncologic outcome of GIST is more likely to be dependent on tumor biology than the type of surgical resection [5]; physical status and CD34 are reported to be prognostic factors [6].

GISTs considered high risk include those >5 cm in diameter and with mitotic count >5 per 50 high power fields (HPFs), those >10 cm in diameter whatever the mitotic count, and those of any size with high mitotic count (>10 per 50 HPFs) [2]. The median survival of patients with GIST measuring 2–5 cm and with mitotic count >5 per 50 HPFs, such as the present case, has been reported to be 49 months [4]. This patient survived for 6 months after surgery without abdominal symptoms. However, careful follow-up is required due to her high mitotic count.

## 4 Conclusion

In conclusion, duodenal GIST can cause duodenojejunal intussusception, and in this rare situation, LR without initial reduction can be performed safely. As demonstrated in this case, even elderly patients can have improved quality of life with limited surgical resection.

### 4.1 Patient perspective

The patient and her family were concerned about whether surgery would adversely affect oral ingestion or other activities of daily living (ADL). Therefore, they agreed to limited surgical resection that would allow oral ingestion. Furthermore, they did not provide consent for a hepatectomy for treatment of the liver metastasis owing to the age of the patient. The surgery was performed with no postoperative complications other than an infection of the surgical site. After the surgery, the patient was able to ingest food, and returned to her normal ADL. The patient was informed about the regular medical follow-up.

## Sources of funding

The research did not receive any specific grant from funding agencies in the public, commercial, or not-for-profit sectors.

## Ethical approval

This case report was approved by the Research Ethics Committee of the Ofuna Chuo Hospital (No. 2019-003).

## Consent

Written informed consent was obtained from the patient for publication of this case report.

## Author contribution

Goshi Fujimoto: Surgeon of the patient’s procedure described in the case report, concept and design of study, acquisition of data, drafting the manuscript, revising the manuscript, and approving the final version of the manuscript.

Shunichi Osada: Assistant during the patient’s surgery described in the case report, revising the manuscript, and approving the final version of the manuscript.

## Registration of research studies

This study was registered as a case report in the UMIN Clinical Trials Registry (https://www.umin.ac.jp/ctr/) with the unique identifying number UMIN000037656.

## Guarantor

Goshi Fujimoto.

## Availability of data and materials

The datasets supporting the conclusions of this article are included within the article.

## Provenance and peer review

Not commissioned, externally peer-reviewed.

## Declaration of Competing Interest

None.
